# Predictive control for the navigation of spherical robots in obstacle-rich environments

**DOI:** 10.1038/s41598-025-96521-6

**Published:** 2025-04-22

**Authors:** Ali Keymasi-Khalaji, Parsa Mokhtari, Fatemeh Bathaei

**Affiliations:** Department of Mechanical Engineering, Faculty of Engineering, Kharazmi University, P.O. Box 15719-14911, Tehran, Iran

**Keywords:** Spherical robot, Predictive control, Obstacle avoidance, Nonholonomic system, trajectory tracking, robot navigation, Engineering, Mechanical engineering

## Abstract

This paper introduces an advanced predictive control algorithm tailored for spherical robots operating within obstacle-dense environments. The proposed strategy employs a sophisticated system model to forecast the robot’s behavior over a defined future time horizon, capitalizing on the strengths of predictive control, which include accelerated convergence time, particularly beneficial in dynamic scenarios. We conduct a comprehensive comparative analysis between the predictive control approach and feedback linearization control, focusing on the robot’s navigation through obstacles. Our findings indicate that the predictive control framework considerably improves the robot’s overall motion performance, leading to shorter response times, enhanced convergence capabilities, and greater resilience when navigating obstacle-dense environments. Furthermore, this approach effectively minimizes control errors, achieving rapid convergence to zero. This research highlights the effectiveness of predictive control in optimizing the agility and accuracy of spherical robots in challenging operational settings. The proposed method represents the first algorithm capable of effectively addressing a comprehensive range of motion tasks for spherical robots. This includes trajectory tracking and both static and dynamic obstacle avoidance with global stability and optimal performance, which are essential for their development.

## Introduction

The rapid advancements in robotic technologies have significantly expanded the scope of mobile robots in various domains^1,2^, including logistics, industrial automation, healthcare, exploration, and disaster response. Among the many types of robotic systems, spherical robots have gained considerable attention due to their unique capabilities. Their compact design, omnidirectional motion, and ability to operate in constrained environments make them ideal for applications such as surveillance^3^, navigation, exploration of hard-to-reach areas, amphibious robotics^4^, and entertainment. However, controlling spherical robots presents significant challenges due to their nonholonomic dynamics, which require precise manipulation of their center of mass. Accurate trajectory tracking, efficient obstacle avoidance, and stable motion control—especially in dynamic environments—remain open challenges in the field.

Spherical robots, as nonholonomic systems, must account for constraints that limit their motion capabilities. These constraints, combined with the dynamic coupling between internal actuators and the robot’s motion, make trajectory tracking a highly nonlinear control problem. Research on spherical robot control has progressed significantly over the years. Early studies, such as^5^, introduced the fundamental equations governing spherical robot dynamics, providing a foundation for understanding their behavior. Building upon this^6^, proposed geometric path-planning algorithms for two-dimensional environments, although these were limited to simplified scenarios. Further exploration in^7^ focused on dynamic analysis and the control methods influencing the robot’s motion in adverse conditions. Later^8^, extended this work by incorporating dynamic models and exploring iterative algorithms for path tracking. While these advancements improved motion precision, they were constrained to specific cases, such as constant-speed motions or predefined trajectories.

Further innovations in spherical robot control have emerged, particularly with the application of advanced control strategies. For instance, higher-order sliding mode control was explored in^9^, resulting in improved trajectory tracking. Similarly^10^, employed the Boltzmann-Hamel approach to derive dynamic equations, enhancing stability and trajectory precision for spherical robots under specific conditions. The path-tracking challenges faced by spherical robots, particularly those with a two-degree-of-freedom pendulum configuration, were critically analyzed in^11^. Observer-based sliding mode control algorithm has also been proposed^12^, demonstrating improved adaptability and robustness scenarios. These approaches, while promising, still face challenges in effectively integrating trajectory tracking with real-time obstacle avoidance in dynamic environments.

Obstacle avoidance remains a critical challenge for spherical robots, especially as they are often deployed in cluttered and unpredictable environments^13^. Obstacle avoidance methods, such as artificial potential fields^14,15^ and discrete-time model predictive control^16^, have demonstrated some success for robotic systems. A broader review of obstacle avoidance algorithms, encompassing classic and contemporary methodologies, was conducted in^17^, assessing the strengths and limitations of various approaches while identifying current trends such as predictive and deep learning strategies.

In addition to spherical robots, significant research has also been dedicated to other non-holonomic robots like wheeled and underwater systems^18–20^. Research on other nonholonomic robotic systems, such as multi-trailer wheeled robots^21,22^ offers valuable insights into addressing similar challenges. Studies on wheeled robots, such as^23] and [24^, introduced kinematic and dynamic modeling techniques that can be applied to spherical robots. For instance, the Taylor series-based approach in^24^ improved path-tracking accuracy for nonlinear systems, while^25] and [16^ demonstrated the effectiveness of Model Predictive Control (MPC) in handling complex maneuvers and constraints. The model predictive controller offers several key advantages that make it highly effective, especially in more complex environments. MPC not only optimizes error signals by predicting future states and adjusting the robot’s trajectory in real time, but it also can handle obstacles with greater precision. Its ability to take into account multiple constraints and future path predictions allows for smoother navigation, which results in faster and more efficient completion of tasks. This makes MPC particularly suitable for environments where quick decision-making and adaptability are crucial. These methodologies, coupled with obstacle avoidance techniques can provide a foundation for improving spherical robot control frameworks.

Recent research has advanced robotic control with methods like Active Disturbance Rejection Control (ADRC), which effectively manages uncertainties and disturbances, improving robustness in applications like mobile robot surveillance^26^. Optimized Model Predictive Control (MPC) has also shown superior precision in UAV trajectory optimization under sensor uncertainties^27^. Additionally, PSO-optimized neural network PID controllers enhance trajectory tracking and disturbance resilience for omnidirectional robots^28^. Artificial intelligence-based optimal control laws are also presented for robotic systems to avoid uncertainties in^29^.

Despite significant advancements, the literature reveals several critical gaps in spherical robot control. Most existing studies focus on either trajectory tracking or obstacle avoidance as isolated problems, failing to address the need for a unified framework that combines both aspects. For example, while^23] and [24^ demonstrated the potential of predictive control, their application to spherical robots with complex dynamics and obstacle-rich environments remains underexplored.

This paper addresses these gaps by proposing a novel control algorithm specifically designed for spherical robots. The proposed framework combines advanced trajectory tracking with obstacle avoidance, leveraging predictive control principles^16^ and obstacle avoidance techniques^14^. By synergistically integrating these components, the proposed algorithm ensures smooth and precise motion control even in complex static and dynamic environments. Unlike traditional methods, the proposed approach accounts for the nonlinear dynamics of spherical robots, enabling more efficient navigation. Extensive case studies are conducted to evaluate the performance of the proposed algorithm under various operational scenarios, including static and dynamic obstacle environments, varying speeds, and complex trajectories. The results demonstrate significant improvements in trajectory tracking, obstacle avoidance, and overall system stability compared to existing methods.

The contributions of this paper are outlined as follows:

- Proposing a novel predictive control algorithm tailored for spherical robots operating in obstacle-dense environments.
- Addressing the critical gap in existing literature by integrating trajectory tracking and obstacle avoidance into a unified control framework for spherical robots.
- Representing the first algorithm capable of effectively addressing a wide range of motion tasks for spherical robots, including trajectory tracking and static/dynamic obstacle avoidance.
- Combining predictive control principles with obstacle avoidance techniques to ensure smooth and precise motion control.
- Ensuring global stability and optimal performance in both static and dynamic environments.

In conclusion, while the field of spherical robot control has advanced significantly, critical challenges remain in achieving a unified approach to trajectory tracking and obstacle avoidance. Existing literature underscores the need for integrated solutions that address the complexities of nonlinear dynamics, stability, and obstacle avoidance. This paper fills this research gap by presenting an innovative control framework that enhances the operational efficiency of spherical robots.

## System description

**Fig. 1:**
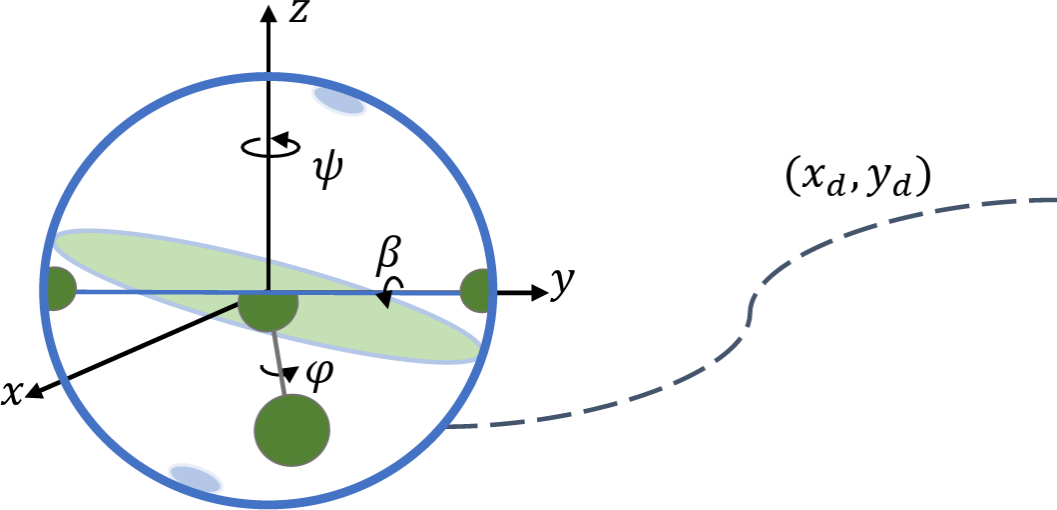
The spherical robot and configuration variables.

The spherical robot operates by adjusting its center of mass as shown in Fig. 1. To effectively model its performance, we consider that a pendulum within the robot’s spherical shell can rotate about two axes. These two rotations include a rotation by angle $\:\beta\:$ around the y-axis and a rotation by angle $\:\phi\:$ around the z-axis. The two angles are a consequence of the torques generated by the two electric motors, which are positioned within the spherical robot along the y and x axes of the sphere. The robot’s position on the Cartesian plane and its orientation are defined by two rotations characterized by the angles β and φ. The equations of motion for the system are derived based on the $\:ZYX$ Euler angles^11^. The spin angle $\:\psi\:$ is a dependent variable of the two angles β and φ, and it determines the orientation of the spherical robot. Consequently, the state vector of the spherical robot is represented by three independent state variables $\:q=(x,\:y,\:\psi\:,\beta\:,\phi\:)$. Thus, the two angular velocities $\:\dot{\beta\:}$ and $\:\dot{\phi\:}$ are the kinematic inputs of the system, controlled using the torques generated by the motors.

System parameters are defined in Table 1.

**Table 1:** System parameters.

Description	Parameter	Description	Parameter
Spherical robot radius	$\:R$	Constraint Matrix	$\:A\left(q\right)$
Robot mass	$\:m$	Jacobian Matrix	$\:S\left(q\right)$
Inertia of the spherical robot around the $\:z$-axis	$\:{I}_{s}$	Mass matrix	$\:M\left(q\right)$
The x-coordinate of the robot’s center of mass	x	The vector for system kinematic control inputs	$\:\varvec{v}$
Linear velocity of the spherical robot’s center of mass along the $\:x$-axis	$\:\dot{x}$	Derivative of the vector of kinematic control inputs	$\:\dot{\varvec{v}}$
The y-coordinate of the robot’s center of mass	y	Coriolis and centripetal forces vector	$\:V(q,\dot{q})$
Linear velocity of the spherical robot’s center of mass along the $\:y$-axis	$\:\dot{y}$	Input torque around the $\:x$-axis	$\:{\tau\:}_{\phi\:}$
Rotation angle of the spherical robot around the $\:x$-axis	$\:\phi\:$	Input torque around the $\:y$-axis	$\:{\tau\:}_{\beta\:}$
Rotation angle of the spherical robot around the $\:y$-axis	$\:\beta\:$	Reference trajectory coordinates	$\:({x}_{d},{y}_{d})$
Rotation angle of the spherical robot around the $\:z$-axis	$\:\psi\:$	Obstacle coordinates	$\:({x}_{obs,i},{y}_{obs,i})$
Angular velocity of the spherical robot around the $\:x$-axis	$\:\dot{\phi\:}$	Distance between the spherical robot and the i-th obstacle	$\:{r}_{obs,\:i}$
Angular velocity of the spherical robot around the $\:y$-axis	$\:\dot{\beta\:}$	Time Horizon	$\:h$
Angular velocity of the spherical robot around the $\:z$-axis	$\:\dot{\psi\:}$	Virtual repulsive force	$\:f$
State vector	$\:q$	Control Parameters of virtual repulsive force functions	$\:{g}_{i},\:n,\:d$
First derivative of state vector	$\:\dot{q}$	Error vector	$\:\varvec{e}$
Second derivative of state vector	$\:\ddot{q}$	Derivative of the error vector	$\:\dot{\varvec{e}}$
input transformation matrix	$\:B\left(q\right)$	MPC cost function	$\:J(u,v)$

## Kinematics model

It is essential to first extract the kinematic and kinetic models of the system. Equation (1) represents the kinematic equations of the spherical robot. These relationships are derived using the rotation matrices obtained from the rotation of the coordinate system attached to the robot, corresponding to the angles $\:\beta\:$ and $\:\phi\:$ around a reference coordinate system, as shown in Fig. 1.

1 $$\left\{\begin{array}{l}\dot{x} = \dot{\beta}\:R\!\cos\!\psi + \dot{\phi}\:R\!\sin\!\psi \cos\!\beta \\\dot{y} = \dot{\beta}\:R\!\sin\!\psi - \dot{\phi}\:R\!\cos\!\psi \cos\!\beta \\\dot{\psi} = \dot{\phi}\:\sin\!\beta\end{array}\right.$$

Equation (1) can be expressed as the product of the constraint matrix $\:A\left(q\right)$ and the system generalized velocity vector $\:\dot{q}$, similar to Eq. 2).

2 $$\:A\left(q\right)\dot{q}=0$$

where

3 $$\:A\left(q\right)=\:\left(\begin{array}{ccc}1&\:0&\:0\\\:0&\:1&\:0\\\:0&\:0&\:1\end{array}\:\:\:\begin{array}{cc}-Rcos\psi\:&\:-Rsin\psi\:cos\beta\:\\\:-Rsin\psi\:&\:Rcos\psi\:cos\beta\:\\\:0&\:-sin\beta\:\end{array}\right)$$

Therefore, the Jacobian matrix S, which is a full-rank matrix, is derived as follows in order to satisfy the relation $\:A\left(q\right)S\left(q\right)\:=\:0$^11^.

4 $$\:S\left(q\right)=\left(\begin{array}{c}\begin{array}{cc}Rcos\psi\:&\:Rsin\psi\:cos\beta\:\\\:Rsin\psi\:&\:-Rcos\psi\:cos\beta\:\\\:0&\:sin\beta\:\end{array}\\\:\begin{array}{cc}1\:\:\:\:\:\:\:\:\:\:\:\:\:\:\:\:\:&\:0\:\:\:\:\:\:\:\\\:0\:\:\:\:\:\:\:\:\:\:\:\:\:\:\:\:\:&\:1\:\:\:\:\:\:\end{array}\end{array}\right)$$

Assuming $$\:v=\left(\begin{array}{c}\dot{\beta\:}\\\:\dot{\phi\:}\end{array}\right)$$ as the system kinematic control inputs vector then the system generalized velocity vector $\:\dot{q}$ can be written as

5 $$\:\dot{q}=S\left(q\right)v$$

## Kinetics model

The kinetics model of the system can be derived using the Lagrange method, as outlined below^11^:

6 $$\:\frac{d}{dt}\frac{\partial\:E}{\partial\:\dot{q}}-\frac{\partial\:E}{\partial\:q}=B\left(q\right)\tau\:-{A}^{T}\left(q\right)\lambda\:$$

where $\:E$ is the system mechanical energy, $\:B\left(q\right)$ is the input transformation matrix, $\:\tau\:={[{\tau}_{\beta}\:\: {\tau}_{\phi}]}^{T}$ is the torque vector^7^. Using Eq. (6), system kinetics model can be obtained as

7 $$\:M\left(q\right)\ddot{q}+V\left(q,\dot{q}\right)-B\left(q\right)\tau\:+{A}^{T}\left(q\right)\lambda\:=0$$

where $\:\lambda\:$ is the vector of Lagrange multipliers and

8 $$\:M\left(q\right)=\left(\begin{array}{ccccc}m&\:0&\:0&\:0&\:0\\\:0&\:m&\:0&\:0&\:0\\\:0&\:0&\:{I}_{s}&\:0&\:-{I}_{s}sin\beta\:\\\:0&\:0&\:0&\:{I}_{s}&\:0\\\:0&\:0&\:-{I}_{s}sin\beta\:&\:0&\:{I}_{s}\end{array}\right)$$

9 $$\:V\left(q,\dot{q}\right)=\left(\begin{array}{c}0\\\:0\\\:-{I}_{s}\dot{\beta\:}\dot{\phi\:}\text{cos}\beta\:\\\:{I}_{s}\dot{\beta\:}\dot{\psi\:}\text{cos}\beta\:\\\:{-I}_{s}\dot{\beta\:}\dot{\psi\:}\text{cos}\beta\:\end{array}\right)$$

10 $$\:B\left(q\right)=\left(\begin{array}{c}\begin{array}{cc}0&\:0\end{array}\\\:\begin{array}{cc}0&\:0\end{array}\\\:\begin{array}{cc}0&\:0\end{array}\\\:\begin{array}{cc}1&\:0\end{array}\\\:\begin{array}{cc}0&\:1\end{array}\end{array}\right)$$

Multiplying $\:{S}^{T}$ on Eq. (7) and using $\:{S}^{T}{A}^{T}\:=0$ yields

Equation (12) is the simplified form of Eq. (11).

11 $$\:{S}^{T}\left(MS\dot{v}\left(t\right)+M\dot{S}v+V\right)={S}^{T}B\left(q\right)\tau\:$$

Similarly we have

12 $$\:{M}_{1}^{{\prime\:}}\left(q\right)\dot{v}+{M}_{2}^{{\prime\:}}\left(q\right)v+\:V\left(q,\dot{q}\right)=B^{\prime}\left(q\right)\tau\:$$

where

13 $$\:\begin{array}{c}{M}_{1}^{{\prime\:}}\left(q\right)={S}^{T}MS\\\:{M}_{2}^{{\prime\:}}\left(q\right)={S}^{T}M\dot{S}\\\:{B}^{{\prime\:}}\left(q\right)={S}^{T}B\left(q\right)\end{array}$$

Alternatively, it can be expressed as

14 $$\tau ={\left( {{B^\prime }\left( q \right)} \right)^{ - 1}}\left\{ {M_{1}^{\prime }\left( q \right)\dot {v}+M_{2}^{\prime }\left( q \right)v+V\left( {q,\dot {q}} \right)} \right\}$$

Finally, the kinetic model of the system can be expressed as

15 $$\dot{v} = M_{1}^{{' - 1}} \left( q \right)\left\{ {B^{\prime } \left( q \right)\tau - M_{2}^{\prime } \left( q \right)v - V\left( {q,\dot{q}} \right)} \right\}$$

## Model predictive control

In this paper, a predictive control approach is proposed for the control of the kinematics of a spherical robot. Predictive control is a technique that has primarily been utilized in the control of electronic systems, power systems, and chemical plants. This method offers a robust framework for controlling complex systems, particularly those characterized by significant time delays and high-order dynamics. Our application of predictive control aims to guide the spherical robot along a reference trajectory while effectively navigating obstacles with minimal response times.

The key distinction of predictive controllers compared to other control methodologies is their ability to forecast future performance at each step. In contrast, many traditional approaches tend to filter out nonlinear factors, mapping the nonlinear model onto a linear framework.

### Kinematic control law

To develop the predictive control law based on the system’s kinematic equations, we will consider the control inputs defined as $\:u=r\dot{\phi\:}\text{c}\text{o}\text{s}\beta\:$ and $\:v=r\dot{\beta\:}$. Consequently, the velocity vector will be expressed as

16 $$\begin{aligned}\left\{\begin{array}{c}\dot{x} \\\dot{y}\end{array}\right\}&=\left[\begin{array}{cc}\sin\psi & \cos\psi \\-\cos\psi & \sin\psi\end{array}\right]\left\{\begin{array}{c}u \\v\end{array}\right\} \\\dot{\psi} &= \dot{\phi}\, \sin\beta\end{aligned}$$

In this approach, the system outputs must be initially predicted using a Taylor Series^23,24,30^. To achieve this, it is essential to first select appropriate candidates to serve as the output functions of the system. In the case of the spherical robot, position errors are selected as the system outputs i.e.

17 $$\{\begin{array}{l}Y_1(t) = e_x \\Y_2(t) = e_y\end{array}$$

where

18 $$\text{\{}\begin{array}{l}e_x = x(t) - x_d(t) \\e_y = y(t) - y_d(t)\end{array}$$

The objective is for $\:{Y}_{i}$ to converge to zero. The Taylor Series representing the future output errors over the time horizon $\:h$ can be expressed as:

19 $$\text{\{}\begin{array}{l}e_x(t+h) = Y_1(t) - h \dot{Y}_1(t) \\e_y(t+h) = Y_2(t) - h \dot{Y}_2(t)\end{array}$$

The initial two terms of the expansion have been selected as an approximation for future system errors in order to facilitate predictions of potential inaccuracies. Substituting (18) into (19) yields

20 $$\text{\{}\begin{array}{l}e_x(t+h) = (x - x_d) - h(\dot{x} - \dot{x}_d) \\e_y(t+h) = (y - y_d) - h(\dot{y} - \dot{y}_d)\end{array}$$

Substituting Eq. (1) into (20) yields

21 $$\text{\{}\begin{array}{l}e_x(t+h) = (x - x_d) + h (u \sin\psi + v \cos\psi - \dot{x}_d) \\e_y(t+h) = (y - y_d) + h (-u \cos\psi + v \sin\psi - \dot{y}_d)\end{array}$$

To evaluate the anticipated performance of the system, we define the cost function J as (24). The function J should incorporate terms that represent the discrepancy between the system’s current position and the desired target. By selecting the cost function as the sum of the squared errors, we ensure that the system’s outputs align closely with the reference outputs.

22 $$\:J(u,v)=\sum\:_{i=x,y}{{k}_{i}e}_{i}^{2}(t+h)$$

Assuming that all tracking errors ($\:{e}_{i}$) hold equal significance, it is established that the weights assigned to all terms in the cost function J are consistent, with the parameters $\:{k}_{x}$ and $\:{k}_{y}$ both set at 1/2. Moreover, in the defined function, the control inputs are considered without weights. By minimizing the function J, the appropriate control inputs over the predicted horizon are obtained. The optimality of the cost function $\:J$ is conditional upon the vanishing of its partial derivatives with respect to the control inputs as

23 $$\text{\{}\begin{array}{l}\dfrac{\partial J}{\partial u} = 0 \\\\\dfrac{\partial J}{\partial v} = 0\end{array}$$

which results in

24 $$\text{\{}\begin{array}{l}\dfrac{\partial e_x}{\partial u} e_x + \dfrac{\partial e_y}{\partial u} e_y = 0 \\\\\dfrac{\partial e_x}{\partial v} e_x + \dfrac{\partial e_y}{\partial v} e_y = 0\end{array}$$

By substituting Eq. (21) into Eq. (24) and solving the resulting equations, we can derive the control inputs for the spherical robot as

25 $$\text{\{} \begin{array}{l}u = \dfrac{1}{h} \{ \cos \psi (y - y_d) - \sin \psi (x - x_d) - h \dot{y}_d \cos \psi + h \dot{x}_d \sin \psi \} \\\\v = \dfrac{1}{h} \{ - \cos \psi (x - x_d) - \sin \psi (y - y_d) + h \dot{x}_d \cos \psi + h \dot{y}_d \sin \psi \}\end{array}$$

As prediction horizon $\:h$ increases, the controller becomes more cautious, emphasizing long-term error minimization and smoother control actions, though excessively large $\:h$ may reduce responsiveness. Conversely, a smaller $\:h$ prioritizes immediate error reduction, leading to more aggressive control but potentially causing overshoots or instability. The chosen value of $\:h$ balances these trade-offs using simulation studies, ensuring prompt responsiveness while maintaining control performance.

### Kinetic control law

In this section, we introduce a kinetic control law that utilizes the Lyapunov method. This approach involves establishing an error function for the kinetic system as

26 $$\:\epsilon\:={v}_{c}-v$$

where $\:{v}_{c}$ is the desired control vector derived from the kinematic control law, and $$\:v=\left[\begin{array}{c}\dot{\beta\:}\\\:\dot{\phi\:}\end{array}\right]$$.

To apply the Lyapunov method, we consider the Lyapunov function candidate, which is defined as a positive definite function as

27 $${V}_{1}=\frac{1}{2}{\epsilon\:}^{T}\epsilon$$

The derivative of the Lyapunov function can be expressed as follows

28 $${\dot{V}}_{1}={\epsilon\:}^{T}\dot{\epsilon\:}$$

By substituting $\:\dot{\epsilon\:}={\dot{v}}_{c}-\dot{v}$ into Eq. (28) yields

29 $${\dot{V}}_{1}={\epsilon\:}^{T}({\dot{v}}_{c}-\dot{v})$$

Substituting from Eq. (15) gives

30 $$\:{\dot{V}}_{1}={\epsilon\:}^{T}\left({\dot{v}}_{c}-{\left({{M}^{{\prime\:}}}_{1}\left(q\right)\right)}^{-1}\left[{B}^{{\prime\:}}\left(q\right)\tau\:-{{M}^{{\prime\:}}}_{2}\left(q\right)v+\:V\left(q,\dot{q}\right)\right]\right)$$

The kinetic control input can be chosen as

31 $$\:\tau\:={\left({B}^{{\prime\:}}\left(q\right)\right)}^{-1}\left\{{{M}^{{\prime\:}}}_{1}\left(q\right){\dot{v}}_{c}+{{M}^{{\prime\:}}}_{2}\left(q\right)v+\:V\left(q,\dot{q}\right)+{{M}^{{\prime\:}}}_{1}\left(q\right)K\epsilon\:\right\}$$

where the matrix $\:K$ is the positive definite gain matrix defined as

32 $$\:K=\left[\begin{array}{cc}{k}_{1}^{{\prime\:}}&\:0\\\:0&\:{k}_{2}^{{\prime\:}}\end{array}\right]$$

By substituting Eq. (32) into (30) results in:

33 $$\:{\dot{V}}_{1}={-\epsilon\:}^{T}K\epsilon\:$$

As can be seen the derivative of the Lyapunov function candidate becomes negative definite for positive definite gain matrices $\:K$, leading to the conclusion that the closed loop system for the spherical robot kinetics model is asymptotically stable.

### Enhanced predictive control to avoid obstacles

The spherical robot has a variety of applications, including search and rescue operations, inspection tasks, and others. In all these contexts, it is essential for the robot to converge to a predetermined trajectory, which is ideally free from obstacles. Consequently, the control system for the spherical robot must be designed to anticipate and enhance its performance in situations where obstacles are present. The aforementioned control strategy applies to scenarios in which the robot can navigate along the assigned trajectory without any obstacles. However, should an obstacle be encountered, the robot must have the capability to adjust its path temporarily before resuming its original path.

At this stage, the system dynamics should be stabilized around the reference trajectories utilizing the proposed control method. Additionally, this approach has been adjusted using a potential field method to prevent collisions with obstacles. The subsequent equations account for the presence of obstacles, with the distance between the spherical robot and each obstacle being determined as follows:

34 $$\:{r}_{obs,i}=\sqrt{{(x-{x}_{obs,i})}^{2}+{(y-{y}_{obs,i})}^{2}}$$

where $\:{x}_{obs,i}$ and $\:{y}_{obs,i}$ are the coordinates of the $\:i$-th obstacle. The virtual repulsive force functions in the $\:x$ and $\:y$ directions are defined as^14,17^:

35 $$\:\left\{\begin{array}{c}{f}_{x,i}={g}_{i}\left(x-{x}_{obs,i}\right)\left(\frac{{r}_{obs,i}^{2}-{d}_{i}^{2}}{{r}_{obs,i}^{n}}\right)\\\:{f}_{y,i}={g}_{i}\left(y-{y}_{obs,i}\right)\left(\frac{{r}_{obs,i}^{2}-{d}_{i}^{2}}{{r}_{obs,i}^{n}}\right)\end{array}\right.$$

In the equations presented above, $\:{g}_{i}$ represents the adjustable parameter, while $\:{d}_{i}$ denotes the diameter or size of the i-th obstacle. The overall resultant repulsive force function, which must be incorporated into the control inputs, is derived as follows:

36 $$\:\left[\begin{array}{c}{f}_{x}\\\:{f}_{y}\end{array}\right]=-1\left(\sum\:_{i=1}^{n}\left[\begin{array}{c}{f}_{x,i}\\\:{f}_{y,i}\end{array}\right]\right)\:\:\:if\:{r}_{i}<{d}_{i}$$

If the reference trajectory is identified as $\:({x}_{d}\left(t\right),{y}_{d}\left(t\right))$, the deviation error from this reference trajectory, considering the presence of obstacles, is expressed as

37 $$\:\left\{\begin{array}{c}{e}_{obs,x}=x-{x}_{d}-{f}_{x}\\{e}_{obs,y}=y-{y}_{d}-{f}_{y}\end{array}\right.$$

By substituting the error equations derived from Eq. (41) into the control law obtained from solving Eq. (26), the MPC control law in the presence of obstacles is derived as shown in Eq. (42).

38 $$\text{\{}\begin{array}{l}u = \frac{1}{h} \left( \cos \psi \left( y - y_d - f_y \right) - \sin \psi \left( x - x_d - f_x \right) - h \dot{y}_d \cos \psi + h \dot{x}_d \sin \psi \right) \\v = \frac{1}{h} \left( - \cos \psi \left( x - x_d - f_x \right) - \sin \psi \left( y - y_d - f_y \right) + h \dot{x}_d \cos \psi + h \dot{y}_d \sin \psi \right)\end{array}$$

## Feedback linearization control

Feedback linearization is a commonly employed technique for controlling nonlinear systems^31^. This approach enables the transformation of a nonlinear system into a linear framework through the selection of an appropriate input. Following this transformation, a control law can be developed for the system. Recently, there has been considerable research focused on the application of feedback linearization in nonholonomic robots.

### Kinematic control law

This section presents the development of a control law for the spherical robot utilizing the existing feedback linearization controller for comparative analysis. The position error of the spherical robot in relation to the desired trajectory, represented by the coordinates $\:\left({x}_{d}\left(t\right),{y}_{d}\left(t\right)\right)$ in the Cartesian coordinate system. This is expressed as $\:{e}_{x}=x\left(t\right)-{x}_{d}\left(t\right)$ and $\:{e}_{y}=y\left(t\right)-{y}_{d}\left(t\right)$.

It is now presumed that the following asymptotically stable error dynamics apply to the closed-loop kinematic control system.

39 $$\:\left\{\begin{array}{c}{\dot{e}}_{x}=-{k}_{1}\text{tanh}{e}_{x}\\\:{\dot{e}}_{y}=-{k}_{2}\text{tanh}{e}_{y}\end{array}\right.$$

Substituting error signals in Eq. (39) results is.

40 $$\:\left\{\begin{array}{c}\dot{x}=-{k}_{1}\text{tanh}{e}_{x}+{\dot{x}}_{d}\left(t\right)\\\:\dot{y}=-{k}_{2}\text{tanh}{e}_{y}+{\dot{y}}_{d}\left(t\right)\end{array}\right.$$

Substituting $$\:\left[\begin{array}{c}\dot{x}\\\:\dot{y}\end{array}\right]$$ from Eq. (1) into (40) yields the following kinematic control input vector for the system.

41 $$\:v={T}^{-1}\left[\begin{array}{c}-{k}_{1}\text{tanh}{e}_{x}+{\dot{x}}_{d}\left(t\right)\\\:-{k}_{2}\text{tanh}{e}_{y}+{\dot{y}}_{d}\left(t\right)\end{array}\right]$$

where.

42 $$\:T=\:\left(\begin{array}{cc}R\text{cos}\psi\:&\:R\text{sin}\psi\:\text{cos}\beta\:\\\:R\text{sin}\psi\:&\:-R\:\text{cos}\psi\:\text{cos}\beta\:\end{array}\right)$$

#### Stability proof

To prove the stability of control law (42), the Lyapunov function candidate is initially considered as.

43 $$\:{V}_{2}=\frac{1}{2}{e}_{x}^{2}+\frac{1}{2}{e}_{y}^{2}$$

By taking the first derivative of the above relation, we obtain the derivative of the Lyapunov function as.

44 $$\:{\dot{V}}_{2}={e}_{x}{\dot{e}}_{x}+{e}_{y}{\dot{e}}_{y}$$

By substituting Eq. (1) into (44), we obtain:

45 $$\:{\dot{V}}_{2}=\left(\dot{\beta\:}Rcos\psi\:+\dot{\phi\:}Rsin\psi\:cos\beta\:-{\dot{x}}_{d}\left(t\right)\right){e}_{x}+\left(\dot{\beta\:}Rsin\psi\:-\dot{\phi\:}Rcos\psi\:cos\beta\:-{\dot{y}}_{d}\left(t\right)\right){e}_{y}$$

Applying Eq. (41) to Eq. (45) yields.

46 $$\:{\dot{V}}_{2}=-{k}_{1}\left|{e}_{x}\right|-{k}_{2}\left|{e}_{y}\right|$$

Equation (46) illustrates the derivative of the Lyapunov function candidate, which is classified as a negative definite function provided that $\:{k}_{1}$ and $\:{k}_{2}$ are positive gains. Consequently, this demonstrates that the selected kinematic control is asymptotically stable.

### Enhanced feedback linearization control to avoid obstacles

If the spherical robot operated by Feedback Linearization Control (FLC) encounters obstacles, it is essential to enhance the robustness of its control law in response to these obstructions. In this context, the virtual force functions associated with the obstacles, based on Eq. (35), can be used.

As a result, the kinematic control law for the spherical robot, taking into account the presence of obstacles in a manner analogous to Eq. (41) and utilizing error signals derived from Eq. (37), is outlined as follows:

47 $$v_{{obs}} = T^{{ - 1}} \left[ {\begin{array}{*{20}c} { - k_{1}^{{\prime}{\prime\:}} \tanh e_{{obs,x}} + \dot{x}_{d} \left( t \right)} \\ { - k_{2}^{{\prime}{\prime\:}} \tanh e_{{obs,y}} + \dot{y}_{d} \left( t \right)} \\ \end{array} } \right]$$

#### Stability proof

The positive definite Lyapunov candidate function $\:{V}_{3}$ is utilized to demonstrate the stability of this control law.

48 $$\:{V}_{3}=\frac{1}{2}{e}_{obs,x}^{2}+\frac{1}{2}{e}_{obs,y}^{2}$$

Thus, the derivative of the Lyapunov function is given by.

49 $$\:{\dot{V}}_{3}={e}_{obs,x}{\dot{e}}_{obs,x}+{e}_{obs,y}{\dot{e}}_{obs,y}$$

Substituting from Eqs. (1) and (37), results in.

50 $$\:{\dot{V}}_{3}=\left(\dot{\beta\:}Rcos\psi\:+\dot{\phi\:}Rsin\psi\:cos\beta\:-{\dot{x}}_{d}\left(t\right)\right){e}_{x}+\left(\dot{\beta\:}Rsin\psi\:-\dot{\phi\:}Rcos\psi\:cos\beta\:-{\dot{y}}_{d}\left(t\right)\right){e}_{y}$$

By substituting from Eq. (47) results in.

51 $$\:{\dot{V}}_{3}=-{k}_{1}^{{\prime}{\prime\:}}{e}_{x}\text{tanh}{e}_{x}-{k}_{2}^{{\prime}{\prime\:}}{e}_{y}\text{t}\text{a}\text{n}\text{h}{e}_{y}$$

It is important to highlight that, in general, the function xtanh(x) (the product of two odd functions) is always positive. By choosing the coefficients $\:{k}_{1}^{{\prime}{\prime\:}}$ and $\:{k}_{2}^{{\prime}{\prime\:}}$ as positive gains, we can ensure that the derivative of the Lyapunov function is negative definite within the space $\:\{{e}_{x},{e}_{y}\}$. This condition guarantees the asymptotic stability of the closed-loop kinematic control system, leading to the convergence of control errors to zero.

## Obtained results

In this section, we conduct a comparison and analysis of Model Predictive Control (MPC) and Feedback Linearization Control (FLC) for the motion control of a spherical robot while considering various reference trajectories and the presence of obstacles.

System parameters and corresponding values are given in Table 2.

**Table 2:** System parameters and corresponding values.

Parameter	Value	Parameter	Value
$\:R$	$\:0.15\:m$	$\:{k}_{1}^{{\prime}{\prime\:}}$	2.0
$\:m$	$\:1.5\:Kg$	$\:{k}_{2}^{{\prime}{\prime\:}}$	1.0
$\:{I}_{s}$	0.014 $\:Kg.{m}^{2}$	$\:{g}_{i}\:(i=1,\dots\:,7)$	2.0
$\:{x}_{d,1}$	$\:\left(1-\left(R+\frac{R}{14}\text{cos}\left(\frac{4t}{5}\right)\right)\right)\text{cos}\left(\frac{t}{5}\right)\:\left(m\right)$	$\:d$	1.7
$\:{y}_{d,1}$	$\:\left(1-\left(R+\frac{R}{14}cos\left(\frac{4t}{5}\right)\right)\right)sin\left(\frac{t}{5}\right)\:\left(m\right)$	$\:n$	6.0
$\:r$	$\:a\:{e}^{b\frac{t}{T}}$ $\:\left(m\right)$	$\:{X}_{obs,1}$	$\:\left(-\text{4,0}\right)\:\left(m\right)$
$\:{x}_{d,2}$	$\:r\text{cos}\left(\frac{t}{T}\right)$ $\:\left(m\right)$	$\:{X}_{obs,2}$	$\:\left(-2,-3\right)\:\left(m\right)$
$\:{y}_{d,2}$	$\:r\text{sin}\left(\frac{t}{T}\right)$ $\:\left(m\right)$	$\:{X}_{obs,3}$	$\:\left(0,-4\right)\:\left(m\right)$
$\:a$	$\:2$	$\:{X}_{obs,4}$	$\:\left(\text{2.5,3.5}\right)\:\left(m\right)$
$\:b$	$\:0.2$	$\:{X}_{obs,5}$	$\:\left(\text{4,0}\right)$ $\:\left(m\right)$
$\:T$	$\:5$	$\:{X}_{obs,6}$	$\:\left(\text{4,0}\right)$ $\:\left(m\right)$
$\:{k}_{1}$	1.0	$\:{X}_{obs,7}$	$\:\left(\text{2,3.5}\right)\:\left(m\right)$
$\:{k}_{2}$	1.0	$\:h$	0.8
$\:{k}_{1}^{{\prime\:}}$	1.2	$\:{k}_{x}$	0.5
$\:{k}_{2}^{{\prime\:}}$	1.0	$\:{k}_{y}$	0.5

**Fig. 2:**
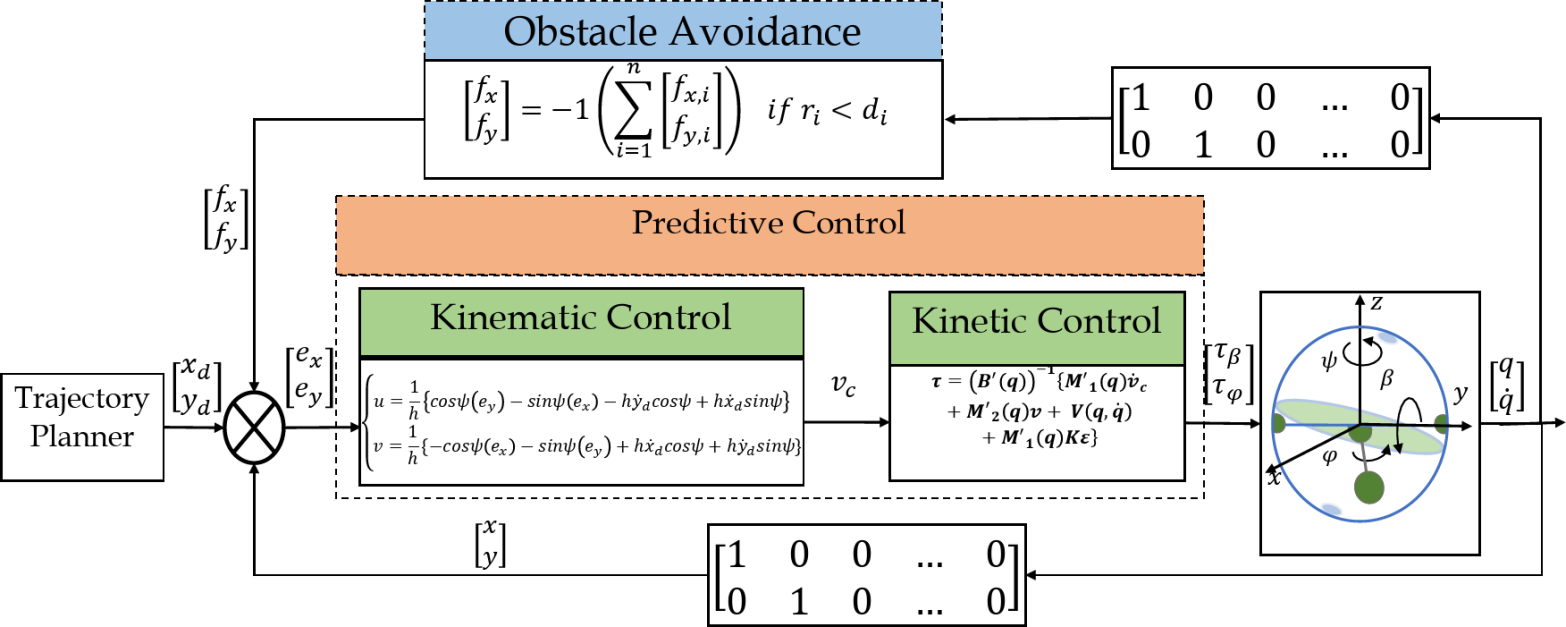
The block diagram for predictive control of the spherical robot.

The block diagram for predictive control and obstacle avoidance of the spherical robot is depicted in Fig. 2.

As illustrated in Fig. 2, the spherical robot tracks the desired trajectory through a control system that integrates both kinematic and dynamic controllers. The kinematic control of the spherical robot is executed utilizing Model Predictive Control (MPC) in one instance and Feedback Linearization Control (FLC) in another. In both scenarios, dynamic control of the spherical robot is governed by a Lyapunov-based approach. Following the implementation of these controllers on the robot’s kinematic system, an analysis and comparison of the robot’s behavior is conducted.

### Case-study 1: trajectory tracking in environments containing static obstacles

**Fig. 3:**
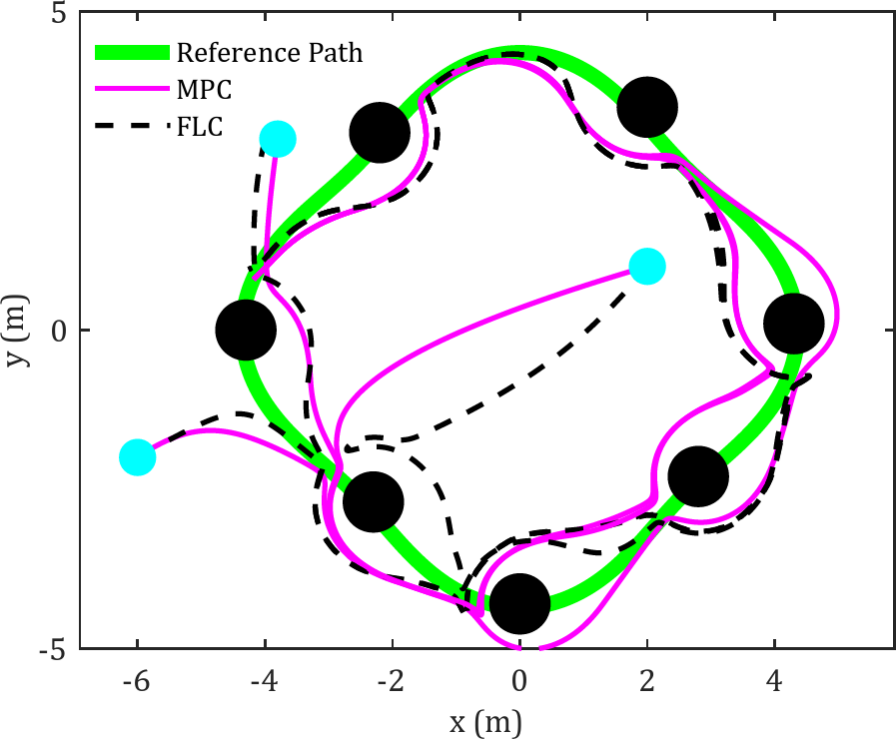
Cartesian path for FLC and MPC in tracking diamond-shaped trajectory in an obstacle-rich environment.

In this case study, six obstacles are positioned along a diamond-shaped trajectory, and the results are illustrated in Fig. 3. The equations representing the reference trajectory are as follows:

52 $$\:\left\{\begin{array}{c}{x}_{d1}=\left(1-\left(R+\frac{R}{14}\text{cos}\left(\frac{4t}{5}\right)\right)\right)\text{cos}\left(\frac{t}{5}\right)\\\:{y}_{d1}=\left(1-\left(R+\frac{R}{14}cos\left(\frac{4t}{5}\right)\right)\right)sin\left(\frac{t}{5}\right)\end{array}\right.$$

The Cartesian coordinates of the obstacles are outlined as follows:

53 $$\:{X}_{\text{1,1}}=\left(-\text{4,0}\right),\:{X}_{\text{1,2}}=\left(-2,-3\right),\:{X}_{\text{1,3}}=\left(0,-4\right),{X}_{\text{1,4}}=\left(\text{2.5,3.5}\right),\:{X}_{\text{1,5}}=\left(\text{4,0}\right),\:{X}_{\text{1,6}}=\left(\text{2,3.5}\right)$$

It is important to highlight that the control parameters have been carefully chosen to ensure system stability and to deliver ideal performance during each control phase.

**Fig. 4:**
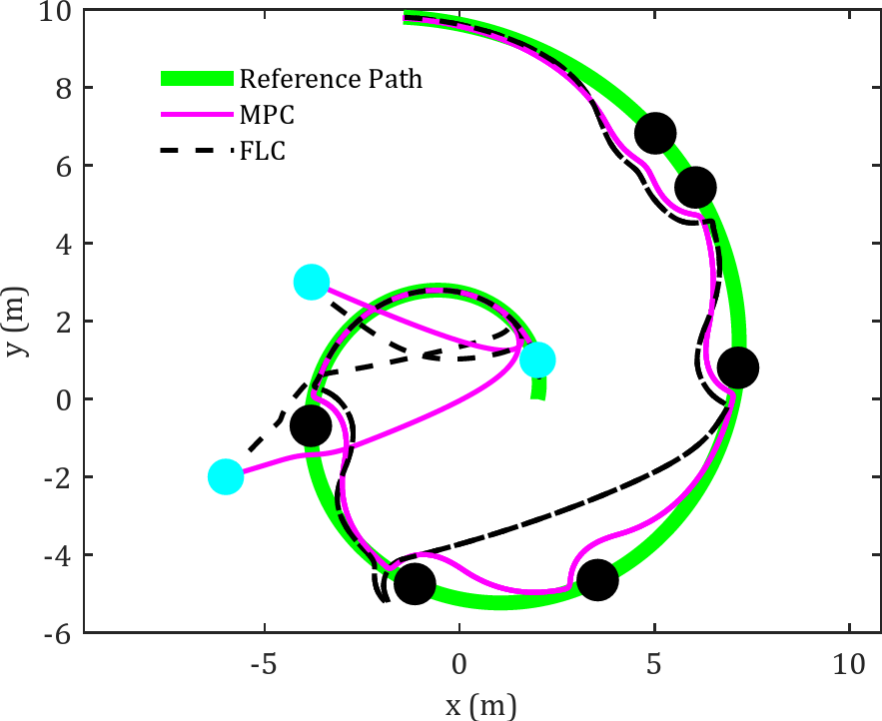
Cartesian path for FLC and MPC in tracking spiral trajectory in an obstacle-rich environment.

Figures 3 and 4 present a comparative analysis of FLC and MPC in terms of their effectiveness in tracking diamond-shaped and spiral trajectories within an obstacle-rich environment.

The reference trajectories are illustrated in green in the figures. The spherical robot initiates motion from multiple starting positions and progressively aligns itself with the reference trajectory. Preliminary observations suggest that the MPC controller enables the robot to reach the planned trajectory more efficiently. Significantly, when faced with obstacles or unexpected changes in the trajectory, the MPC controller displays enhanced accuracy in converging to the desired trajectory. Under MPC control, the spherical robot shows a more proficient response to obstacles, allowing for closer proximity to them and a reduced time to achieve the desired trajectory compared to the FLC.

Figure 4 illustrates the results of the spherical robot’s motion control using MPC and FLC along a predefined spiral trajectory. This reference trajectory is configured as follows

54 $$\:\left\{\begin{array}{c}\begin{array}{c}{x}_{d2}=r\text{cos}\left(\frac{t}{T}\right)\\{y}_{d2}=r\text{sin}\left(\frac{t}{T}\right)\end{array}\\\:r=a\:{e}^{\frac{bt}{T}}\:\:\:\:\:\:\:\:\:\:\:\end{array}\right.$$

The spherical robot starts its motion from three distinct initial points, successfully converging toward the reference trajectory while utilizing two different control methods. An initial evaluation of the trajectory exhibited by the spherical robot under the FLC indicates that the robot converges to the desired trajectory with reasonable accuracy. However, in the presence of obstacles along the trajectory, the performance of the FLC controller deteriorates. In contrast, the MPC controller demonstrates a considerable enhancement in the robot’s capability to follow the reference trajectory. This distinction between the two controllers becomes particularly evident when passing the second and third obstacles. The results further confirm the MPC controller’s superior rate of convergence to the trajectory compared to the FLC controller.

**Fig. 5:**
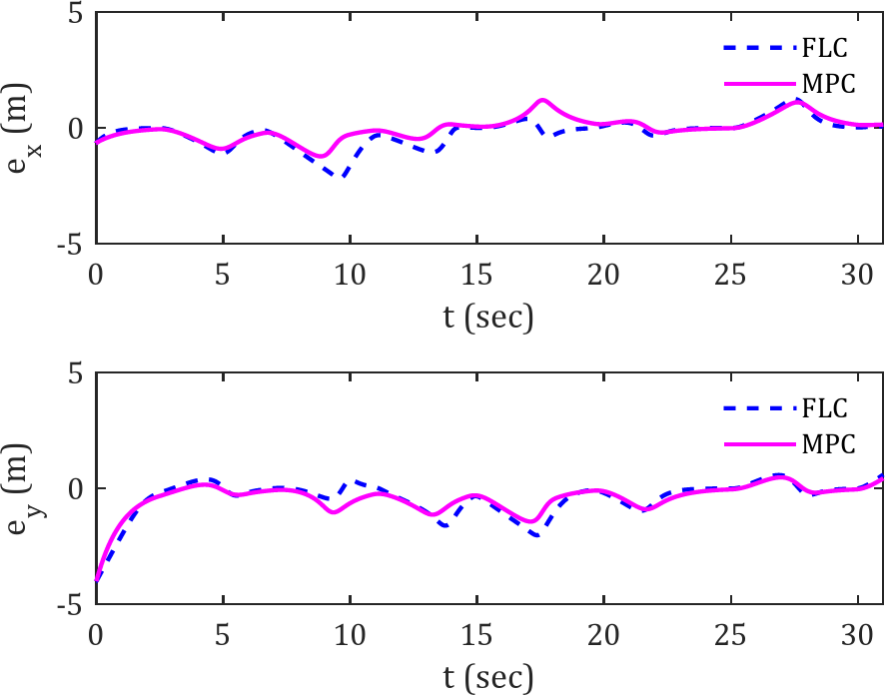
Error signals for tracking diamond-shaped reference trajectory along the x and y axes in the presence of obstacles.

While the path taken by the spherical robot does not enable definitive conclusions regarding temporal variations, the error plots provide validation for these observations. The error plots $\:{e}_{x}\left(t\right)$ and $\:{e}_{y}\left(t\right)$ for tracking the diamond-shaped reference trajectory are presented in Fig. 5.

Two key points can be discussed here. First, the deviation error is smaller for the MPC controller compared to the FLC controller, indicating that the MPC controller is more resilient to obstacles, allowing the spherical robot to maintain closer proximity to the desired trajectory without excessive deviation when obstacles are present.

Another advantage of the MPC controller over the FLC controller is its faster convergence to the trajectory. This is observed from the shorter distance and time for the error to reach zero in the MPC compared to the FLC. This high trajectory-following accuracy can be attributed to the inherent nature of the MPC controller, which operates based on a forward-looking prediction horizon. In this regard, the appropriate selection of parameter h becomes crucial. For instance, when encountering an obstacle, the MPC controller detects the obstacle’s clearance more quickly than the FLC controller, enabling a faster return to the trajectory. This is because it continuously predicts its trajectory in short, sequential time intervals, optimizing the control inputs.

**Fig. 6:**
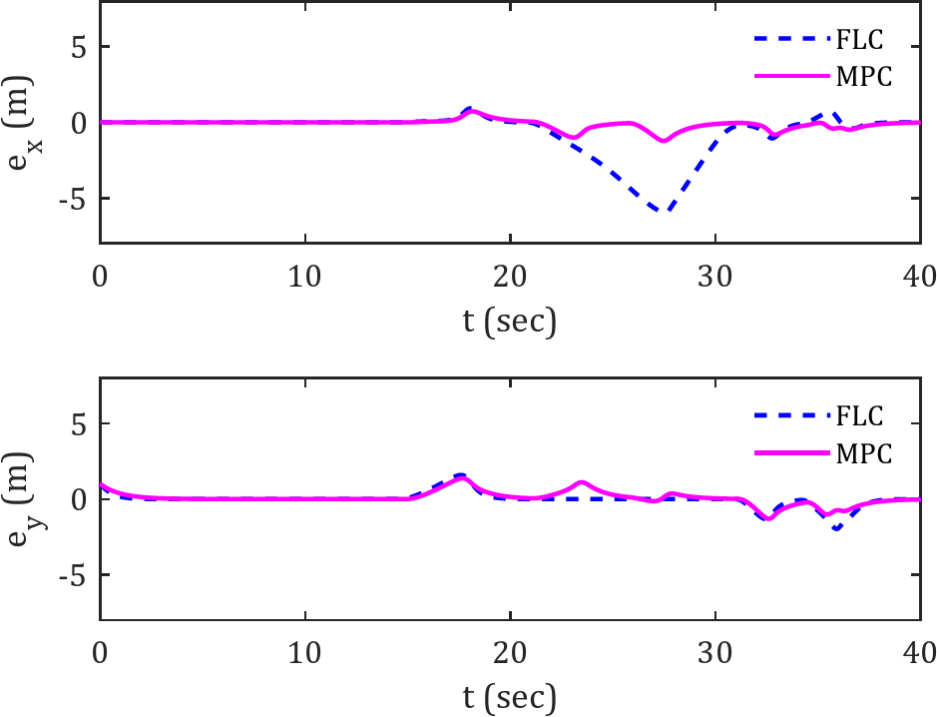
Error signals for tracking spiral reference trajectory along the x and y axes in the presence of obstacles.

Figure 6 display the deviation error plots for the spiral reference trajectory. Observing the FLC and MPC controller plots, particularly within the 20–30 s time interval, reveals the superiority of the MPC controller in rapidly reducing the error. In other words, the MPC controller tends to keep the spherical robot at a lower deviation from the reference trajectory compared to the FLC controller. This difference between the two controllers becomes more pronounced in the presence of obstacles, as evidenced by the nearly identical behavior of the black and pink curves during the initial 15 s, supporting this assertion.

**Fig. 7:**
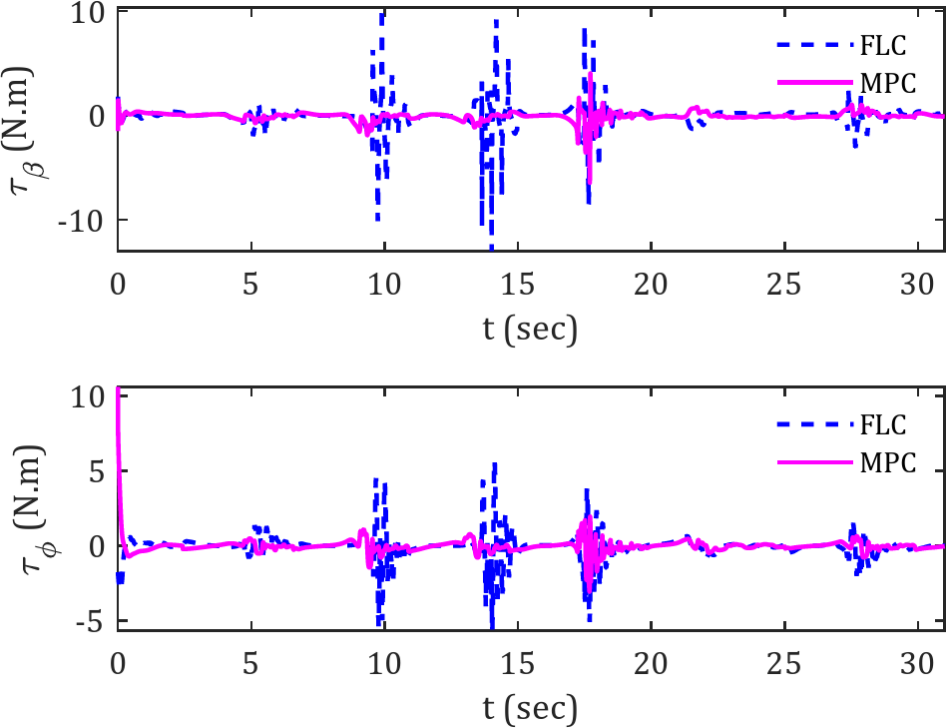
Actuator torques for tracking diamond-shaped reference trajectory in the presence of obstacles.

Figure 7 illustrates the applied torques in the φ and β channels for the diamond-shaped reference trajectory. This indicates a significant difference in torque variations, as the magnitude of overshoots when encountering obstacles is considerably higher with the FLC controller than with the MPC controller.

High torque variations are considered an unfavorable characteristic, as they can lead to operational difficulties for the actuators of the spherical robot that are tasked with generating torque. Consequently, while the movement of the spherical robot may seem achievable in proximity to the reference trajectory, implementing this effectively in practice would present significant challenges.

In analyzing Fig. 6, particularly during the first 20 s interval, a noticeable difference in torque values between the MPC and FLC is evident. A comparison of plots 6 and 7 reveals that when the spherical robot, under FLC control, attempts to correct its deviation error, it exerts larger torque values. Conversely, the MPC-controlled spherical robot not only sustains a lower deviation error but also converges to the reference trajectory with smaller torque applications. This observation highlights a potential disadvantage of the FLC controller.

**Fig. 8:**
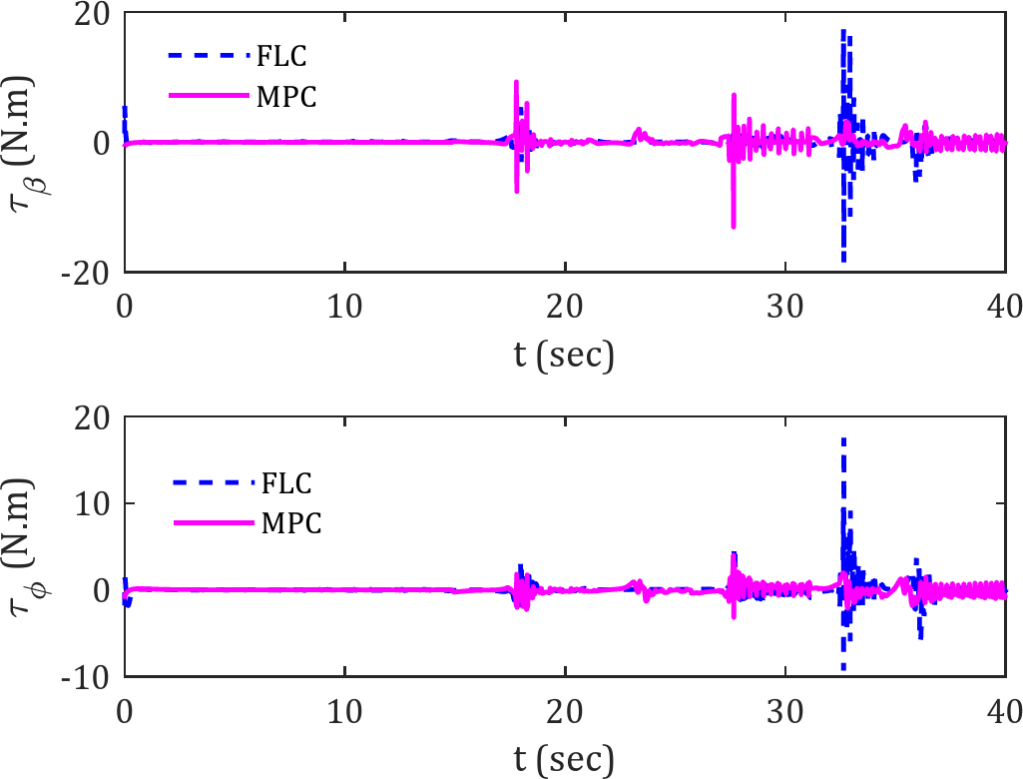
Actuator torques for tracking spiral reference trajectory in the presence of obstacles.

Figure 8 demonstrates the torque variations of the spherical robot when utilizing the two introduced controllers for tracking a spiral trajectory, thereby reaffirming previous assertions.

**Table 3:** Numerical comparison of FLC and MPC algorithms for error signals and control inputs in tracking diamond-shaped trajectory.

Averaging criterion	$\tau _{\beta }^{{FLC}}$	$\tau _{\varphi }^{{FLC}}$	$\tau _{\beta }^{{{\text{MPC}}}}$	$\tau _{\varphi }^{{{\text{MPC}}}}$	$e_{x}^{{FLC}}$	$e_{y}^{{FLC}}$	$e_{x}^{{{\text{MPC}}}}$	$e_{y}^{{{\text{MPC}}}}$
RMS	4.27	0.90	0.87	0.28	0.73	1.21	0.19	0.57
MAE	1.38	0.41	0.17	0.12	0.49	0.78	0.14	0.25
MSE	18.27	0.81	0.75	0.08	0.53	1.46	0.04	0.33

**Table 4:** Numerical comparison of FLC and MPC algorithms for error signals and control inputs in tracking spiral trajectory.

Averaging criterion	$\tau _{\beta }^{{FLC}}$	$\tau _{\varphi }^{{FLC}}$	$\tau _{\beta }^{{{\text{MPC}}}}$	$\tau _{\varphi }^{{{\text{MPC}}}}$	$e_{x}^{{FLC}}$	$e_{y}^{{FLC}}$	$e_{x}^{{{\text{MPC}}}}$	$e_{y}^{{{\text{MPC}}}}$
RMS	0.91	0.80	0.63	0.47	1.70	0.49	0.33	0.45
MAE	0.31	0.30	0.28	0.26	0.80	0.22	0.19	0.28
MSE	0.83	0.64	0.39	0.22	2.89	0.24	0.11	0.20

As shown in Tables 3 and 4, the statistical analysis demonstrates that, when comparing Model Predictive Control (MPC) and Feedback Linearization Control (FLC), the error metrics (RMS, MAE, and MSE) are consistently lower for the MPC method. These results are visually represented in the corresponding graphs, where it is evident that MPC achieves a significantly smaller deviation from the reference path. The numerical data further supports this observation, confirming that MPC yields lower error values across all three metrics. This suggests that MPC is more effective in minimizing the error in the system’s performance.

An important aspect to consider is that, although the control inputs in the FLC method are higher on average compared to MPC, the MPC method achieves lower error values with these relatively smaller control inputs. This observation indicates that MPC is able to produce more accurate results with less control effort, highlighting its efficiency in optimizing the control process.

### Case-study 2: trajectory tracking in environments containing moving obstacles

**Fig. 9:**
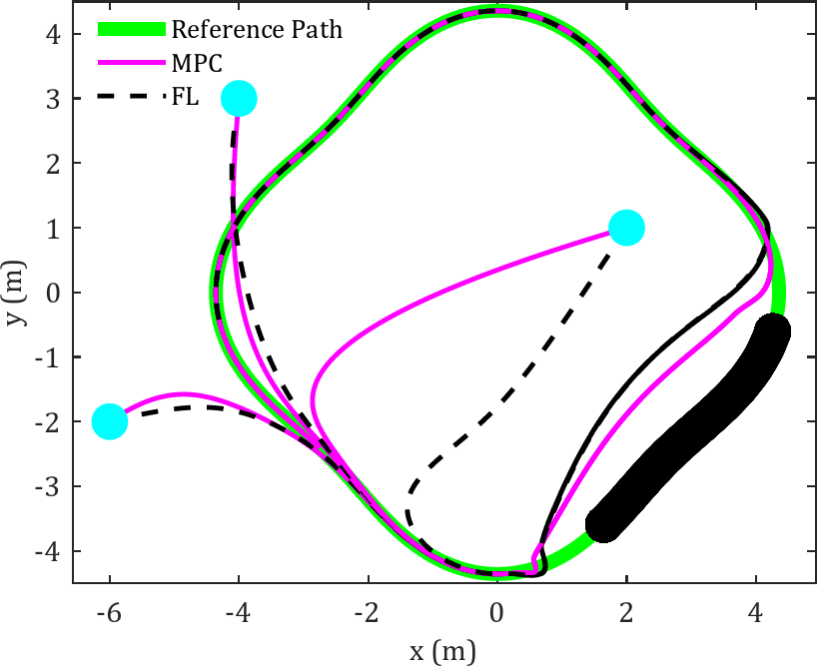
Cartesian path for FLC and MPC in tracking diamond-shaped trajectory in the presence of moving obstacle.

This section aims to assess the precision of the spherical robot’s control system in response to moving obstacles. Figure 9 presents the trajectory tracking diagram associated with following the diamond-shaped trajectory. The moving obstacle, indicated in black on the diagram, attached to the specified trajectory. Regarding the dynamic obstacle avoidance implementation, in the current method, dynamic obstacles and their trajectories are predefined for the control system. This means the predictive control operates under the assumption that information about obstacle positions and trajectories is known in advance. While this approach allows for effective obstacle avoidance in controlled scenarios, it does not currently handle unknown movements or unknown obstacle trajectories in real-time.

As can be seen, obtained results indicate that the spherical robot, under MPC control, converges to the reference trajectory with less error compared to the FLC controller after passing the obstacle. At the start of the motion, the difference between the two controllers is noticeably evident. As expected, examining different starting points for the spherical robot reveals that MPC achieves convergence to the trajectory in a shorter time compared to FLC. Another important aspect in analyzing the spherical robot’s motion under the influence of the two controllers is the reduced deviation of the robot when passing near the moving obstacle under MPC control. This means that, when controlled by FLC, the spherical robot maintains a greater lateral distance from obstacles.

In this section, the appropriate selection of the control parameters $\:d$ and $\:n$ in both controllers is crucial, as these parameters affect the deviation location and the extent of the spherical robot’s deviation from the trajectory.

**Fig. 10:**
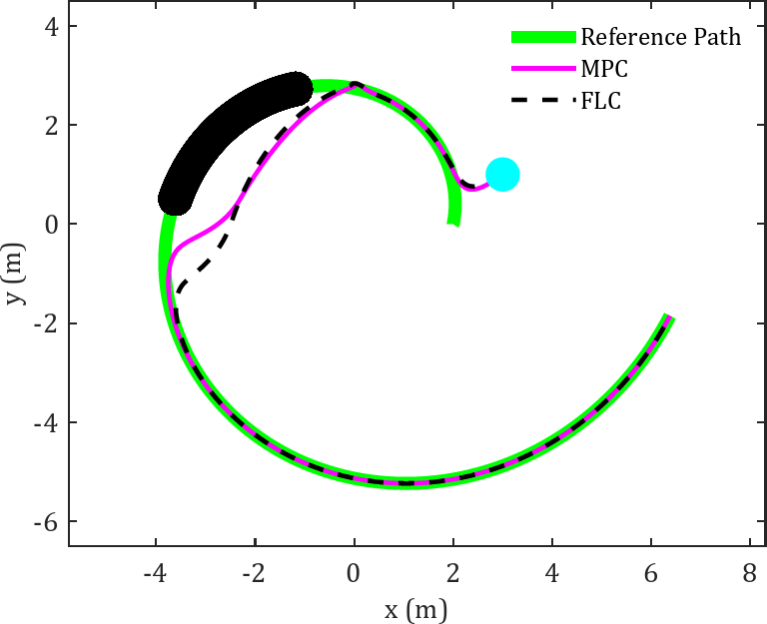
Cartesian path for FLC and MPC in tracking spiral trajectory in the presence of moving obstacle.

The above results are also applicable to another candidate trajectory. Figure 10 depicts the trajectory of the spherical robot along the spiral trajectory under the specified controllers. In this diagram, the faster return of the robot after avoiding the obstacle when moving with the MPC controller, compared to the FLC controller, is clearly observed.

**Fig. 11:**
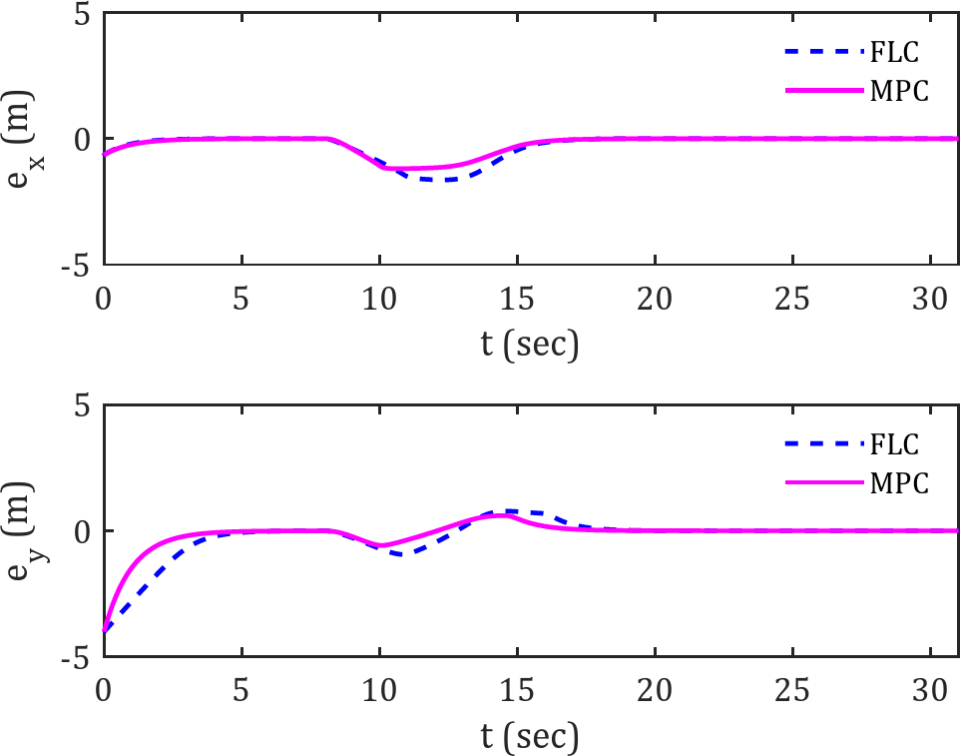
Error signals in tracking diamond-shaped trajectory along the x and y axes in the presence of moving obstacles.

Figure 11 illustrate the tracking error variations of the spherical robot over time in the $\:x$ and $\:y$ directions along the diamond-shaped trajectory.

The results indicate that the deviation error from the trajectory remains in vicinity of zero throughout the trajectory, except for the period when the spherical robot is in proximity to the moving obstacle. The superior resilience of the MPC controller compared to the FLC in handling obstacles can be inferred from the trajectory deviation error graphs. Under MPC control, the spherical robot can maintain an optimal distance to the reference trajectory compared to FLC. The rapid convergence of the graph to zero and the lower trajectory error relative to the desired trajectory under MPC control confirm this claim.

**Fig. 12:**
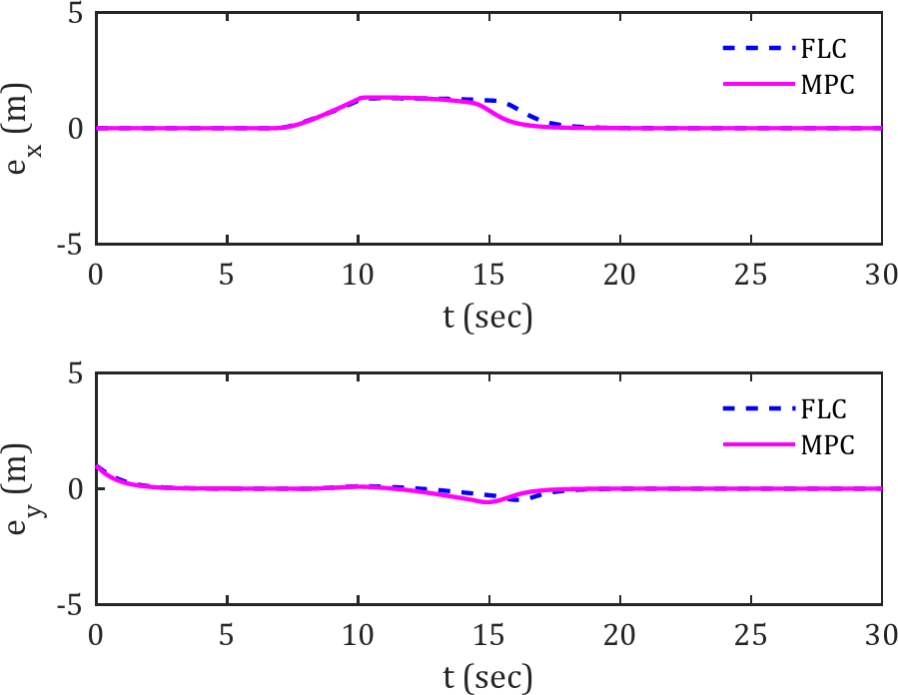
Error signals in tracking spiral trajectory along the x and y axes in the presence of moving obstacles.

Figure 12 illustrates the trajectory deviation error of the spherical robot while tracking the spiral reference trajectory in the presence of a moving obstacle.

This further confirm the claim of the MPC controller’s superior performance compared to FLC when encountering moving obstacles. During the time interval of 7–20 s, which includes the interaction with the moving obstacle, the lower error in the MPC graph, as well as its faster convergence to zero compared to the FLC, is evident.

**Table 5:** Numerical comparison of FLC and MPC algorithms for error signals and control inputs in tracking diamond-shaped trajectory in the presence of moving obstacle.

Averaging criterion	$\tau _{\beta }^{{FLC}}$	$\tau _{\varphi }^{{FLC}}$	$\tau _{\beta }^{{{\text{MPC}}}}$	$\tau _{\varphi }^{{{\text{MPC}}}}$	$e_{x}^{{FLC}}$	$e_{y}^{{FLC}}$	$e_{x}^{{{\text{MPC}}}}$	$e_{y}^{{{\text{MPC}}}}$
RMS	0.46	0.19	0.21	0.14	0.90	1.71	0.41	0.60
MAE	0.14	0.11	0.10	0.09	0.55	1.04	0.20	0.24
MSE	0.21	0.04	0.04	0.02	0.82	2.91	0.17	0.36

The statistical analysis (Table 5) and graphical results demonstrate that Model Predictive Control (MPC) consistently surpasses Feedback Linearization Control (FLC) in performance, exhibiting lower RMS, MAE, and MSE error values and smaller deviations from the reference trajectory. Notably, MPC achieves superior accuracy with reduced control effort compared to FLC, underscoring its efficiency in optimizing system performance. These findings highlight MPC’s effectiveness in minimizing errors while maintaining lower control inputs.

## Conclusion

This paper introduced a predictive control framework tailored for spherical robots, addressing the critical challenges of trajectory tracking and obstacle avoidance in dynamic and static obstacle-dense environments. By utilizing predictive control ability to forecast future states and optimize control inputs, the proposed approach demonstrated superior performance compared to traditional feedback linearization methods. The framework achieved faster response times (on average, less than 10%), reduced control errors (on average, less than 40%), improved settling capabilities (on average, less than 10%), and enhanced adaptability to avoid obstacles. In this study, we conducted a thorough comparison by placing the spherical robot on various trajectories containing both static and dynamic obstacles to evaluate the controllers. Obtained results and case studies highlight the framework’s effectiveness in ensuring precise navigation, robust obstacle avoidance, and overall system stability. This unified approach successfully integrates trajectory tracking with obstacle avoidance, overcoming limitations in existing methods and providing a reliable solution for practical applications. This work advances the state of the art in spherical robot control. It offers an efficient solution for navigating challenging environments, contributing valuable insights to the field of autonomous robotic systems. Future research may focus on expanding the framework with advanced machine learning-based prediction models to further enhance its performance in highly dynamic, uncertain and unpredictable environments. Key areas of exploration include the extension to multi-robot systems, real-world deployment, enhanced dynamic obstacle avoidance, and the examination of three-dimensional environments.

## Data Availability

The datasets used and/or analysed during the current study available from the corresponding author on reasonable request.
